# Determinants of tuberculosis infection and disease in paediatric patients in the Czech Republic after tuberculosis exposure

**DOI:** 10.1093/inthealth/ihaf151

**Published:** 2025-12-22

**Authors:** Luboš Bača, Martin Magner, Karolína Doležalová

**Affiliations:** Department of Paediatrics of the First Faculty of Medicine, Charles University, Thomayer University Hospital, Vídeňská 800, 140 59, Prague 4, Czech Republic; Department of Paediatrics and Inheritd Metabolic Disorders of the First Faculty of Medicine, Charles University, General University Hospital, Ke Karlovu 2, 128 08, Prague 2, Czech Republic; Department of Paediatrics of the First Faculty of Medicine, Charles University, Thomayer University Hospital, Vídeňská 800, 140 59, Prague 4, Czech Republic

**Keywords:** child, computed tomography, contact tracing, tuberculosis, tuberculosis infection, Ukrainian refugee

## Abstract

**Background:**

*Mycobacterium tuberculosis* is a transmissible pathogen most commonly acquired through contact with an infectious individual. Early initiation of tuberculosis (TB) preventive treatment (TPT) in high-risk populations can substantially reduce the risk of TB infection (TBI) and/or progression to active disease. Our objectives were to identify risk factors for children and adolescents developing TBI and TB after exposure and to evaluate the diagnostic utility of chest computed tomography (CT).

**Methods:**

A 6.5-year retrospective cohort study was conducted at a single centre in the Czech Republic to assess risk factors for TBI and TB in children and adolescents (0–18 y).

**Results:**

Of 335 children included in the study (median age 6.85 y), TBI was diagnosed in 83 (25%) and TB in 39 (12%). Chest CT in 45 high-risk contacts identified TB in an additional 13 participants. The key TB risk factor was index patients having smear-positive TB (odds ratio [OR] 11.35), while older age of contacts (OR 0.83) and non-household exposure (OR 0.23) had a lower risk of TB. TBI risk increased with age (OR 1.21), index smear-positive patients (OR 4.66) and when the father was the index patient (OR 2.91). Ukrainian refugee children had higher risks of TB (relative risk 3.4). Adherence to TPT in those who qualified was good.

**Conclusions:**

Risk factors for TBI and TB were identified, including children being from Ukraine. Chest CT may aid in early diagnosis of TB.

## Introduction

Contact investigation, also known as contact tracing, is viewed as a fundamental element of the End TB Strategy.^1^ When implemented effectively, contact investigation enables the prompt commencement of preventive treatment in high-risk individuals, thereby reducing the likelihood of progression to tuberculosis infection (TBI) or tuberculosis disease (TB). In addition, the timely isolation and treatment of people with infectious TB is crucial to reduce transmission of infection.^2^ The efficacy of contact tracing is influenced by people’s cooperation with the recommended health system measures and procedures.^3^ The analysis of risk factors for TBI and TB may assist in improving contact tracing procedures.

To date, no study has examined the contact tracing system for children exposed to TB in the Czech Republic, which is considered a low-burden TB country.^4^ In the course of the present study, the geopolitical situation in Europe underwent a significant change, precipitated by two major developments: the Russian invasion of Ukraine and a substantial influx of migrants. By the end of June 2023, >530 000 Ukrainians had been granted temporary protection status in the Czech Republic,^5^ although the actual number of migrants is likely considerably higher. Given the high incidence of TB and drug-resistant TB (DR-TB) in Ukraine,^6^ this influx has had a substantial impact on the incidence of TB and DR-TB since 2022.^7^

In addition, the diagnostic approach to identifying early TB disease was modified during the course of the study. The decision was taken to initiate a more extensive indication of chest computed tomography (CT) of the thorax in instances where an inconclusive chest X-ray (CXR) had been obtained.

The objective of this study was to characterise the outcome of contact tracing in children and adolescents, identify risk factors for TBI and TB overall, compare Ukrainian refugee children—coming from a high-TB-burden setting—to other children from the Czech Republic and evaluate the possible role of chest CT in the diagnosis of early TB in child contacts.

## Methods

We conducted a single-centre descriptive retrospective cohort study of children and adolescents exposed to TB in the Czech Republic. Initial examinations were performed between January 2018 and December 2023, with subsequent follow-ups completed by March 2024. For participants with non-infected status, the planned observation period was at least 3 months, and for TBI participants, at least 6 months. The study population included children who underwent their initial examinations at our centre (Thomayer University Hospital, Prague), as well as those who were referred to our centre by external physicians for further evaluation after an initial examination. All data were obtained from electronic medical records of participants seen in our department. The data were anonymized and processed in accordance with the approval of the Ethics Committee of the Institute for Clinical and Experimental Medicine and the Thomayer University Hospital (22654/21 and 25455/21). A waiver of individual informed consent was obtained because of the retrospective nature of the study. The inclusion criteria were individuals <19 y of age with and International Classification of Diseases code Z20.1 (contact with and [suspected] exposure to TB). Children and adolescents exposed to culture-negative TB or extrapulmonary TB cases were excluded from the cohort. Participants were characterised by age, sex, Bacille Calmette-Guérin (BCG) vaccination status, exposure setting (household or non-household), familial relationship to the index patient and the index patient’s smear microscopy, microbiological and drug susceptibility results.

In the Czech Republic, the protocol for the management of children and adolescents after contact with TB adheres to the national guidelines from 2021.^8^ The initial examination consists of a medical history, physical examination, CXR, interferon-gamma release assay (IGRA) test and/or tuberculin skin test (TST) for each child >1 month of age. According to recommendations for the diagnosis of TB, chest CT is indicated in children <10 y of age with definite epidemiological contact and a positive IGRA or TST.^9^

TB preventive treatment (TPT) is recommended for high-risk children (<5 y of age or those who are immunosuppressed), regardless of TST/IGRA results, as well as for older children and adolescents with confirmed TBI. Recommended regimens include 3 months of daily isoniazid plus rifampicin (3HR), 4 months of daily rifampicin (4R) or 6 months of daily isoniazid (6H).^9^

TB was defined as a clinical condition characterized by imaging findings consistent with TB or microbiological confirmation of *Mycobacterium tuberculosis* complex in conjunction with a positive TST and/or IGRA. TBI was defined as a positive TST and/or IGRA without clinical symptoms, abnormal imaging findings or microbiological evidence. Non-infected status was defined as a negative TST and/or IGRA, with no clinical symptoms suggestive of TB, no pathological findings on imaging and no microbiological evidence of TB disease.

TST positivity was defined as an induration >10 mm at 48–72 h in children with prior BCG vaccination and >5 mm in children without BCG vaccination. IGRA positivity was determined according to the manufacturer’s instructions for QuantiFERON-TB Gold Plus (Qiagen, Santa Clarita, CA, USA). Refugee status was defined as children born in Ukraine who migrated to the Czech Republic after 24 February 2022.

### Statistical analysis

Binary logistic regression was used to examine potential risk factors for TBI plus TB (as well as for TBI and TB separately). For the categorical variable sex, the reference category was ‘male’, for BCG response it was ‘no BCG’, for contact frequency it was ‘household’, for index type it was ‘smear negative’, for contact sensitivity it was ‘sensitive’ and for relationship it was ‘mother’. The variable of age was treated as a continuous variable. A p-value <0.05 was considered to be statistically significant. Data were analysed with SPSS (IBM SPSS Statistics 23, Armonk, NY, USA) and Excel 2024 (Microsoft, Redmond, WA, USA). The methodology of the study is consistent with the Strengthening the Reporting of Observational Studies in Epidemiology protocol.

## Results

**Figure 1.**
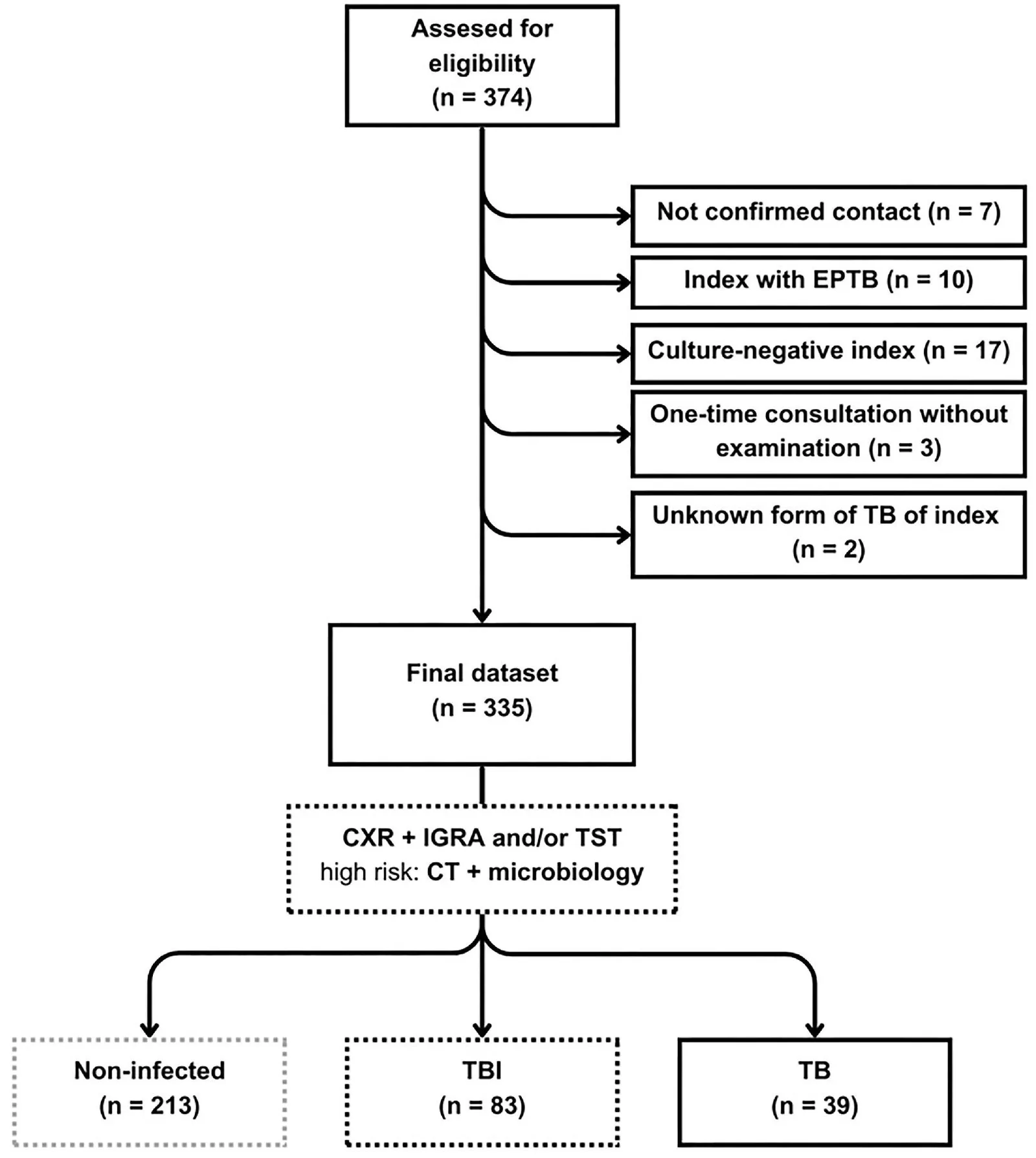
The diagram illustrates the cohort of excluded participants, the diagnostic approach to participants included in the final dataset (outlined with a dotted line) and the final outcome after the follow-up period. EPTB; extrapulmonary TB.

Of 374 identified child and adolescent contacts, 272 (72.7%) were primarily evaluated at our centre and 102 (27.3%) were referred from other facilities. Thirty-nine (10.4%) children were excluded (reasons shown in Figure 1), leaving 335 contacts included in the study.

Among the 335 participants, 330 (98.5%) underwent initial testing with either IGRA or TST. Both tests were performed in 202 children, 113 underwent TST only and 15 underwent IGRA only. Discrepancies between IGRA and TST results were observed in 17 children: 13 had IGRA-positive/TST-negative results and 4 had IGRA-negative/TST-positive results. A total of 76 (22.7%) participants underwent chest CT. Among the 45 children with a normal CXR, 13 demonstrated CT findings suggestive of TB, predominantly lymphadenopathy. Microbiological specimens were obtained in 76 (22.7%) children; of these, 10 were positive for *M. tuberculosis* complex and all were susceptible to first-line anti-TB drugs. TPT was indicated in 133 children, of whom 127 (95.5%) initiated treatment and 112 (84.2%) completed it. After a median follow-up period of 119 days (interquartile range 64.5–203.5), 213 of 335 children (63.6%) were evaluated as non-infected, 83 (24.8%) as TBI and 39 (11.6%) as TB.

Risk factors for TB, TBI and combined TB/TBI were analysed. For TB, the only statistically significant risk factor was smear positivity of the index patient (odds ratio [OR] 11.35 [95% confidence interval {CI} 2.238 to 57.583], p=0.003). Lower risk was associated with older age (OR 0.839 [95% CI 0.742 to 0.950], p=0.005; each additional year reduced the odds of TB by 16%) and exposure outside the household (OR 0.231 [95% CI 0.054 to 0.994], p=0.05). For TBI, significant risk factors included smear-positive index patients (OR 4.662 [95% CI 1.916 to 11.343], p=0.001), having the father as the index patient (OR 2.910 [95% CI 1.073 to 7.889], p=0.04) and increasing age (OR 1.121 [95% CI 1.030 to 1.220], p=0.008). For combined TB/TBI, risk factors were smear-positive index patients (OR 9.487 [95% CI 3.958 to 22.732], p<0.001), exposure to drug-resistant TB strains (OR 4.034 [95% CI 1.045 to 15.564], p=0.04), having a sibling as the index patient (OR 12.367 [95% CI 1.862 to 82.142], p=0.009) and having a father as the index patient (OR 6.127 [95% CI 1.970 to 19.058], p=0.002). Exposure outside the household was again protective (OR 0.362 [95% CI 0.141 to 0.928], p=0.03).

The evaluation of BCG vaccination and the risk of developing TB or TBI in our cohort did not yield a statistically significant result. A summary of risk factors is shown in Table 1, with full statistical details provided in the supplementary appendix.

**Table 1.** Demographic and clinical characteristics of participants at the end of the follow-up period according to status non-infected, TBI and TB.

Characteristics	Total	Non-infected	TBI	TB
Patients, n (%)	335 (100)	213/335 (64)	83/335 (25)	39/335 (12)
Age (years), median (IQR)	6.85 (3.04–11.88)	6.59 (2.59–12.80)	8.99 (5.48–11.59)	3.08 (2.11–7.26)
Sex, n/N (%)				
Male	156/335 (47)	94/213 (44)	41/83 (50)	21/39 (54)
Female	179/335 (53)	119/213 (56)	42/83 (51)	18/39 (46)
BCG vaccination status, n/N (%)				
Yes	172/331 (52)	112/211 (53)	47/81 (58)	13/39 (33)
No	159/331 (48)	99/211 (47)	34/81 (42)	26/39 (67)
Household exposure, n/N (%)				
Yes	135/268 (50)	64/167 (38)	43/65 (66)	28/36 (78)
No	133/268 (50)	103/167 (62)	22/65 (34)	8/36 (22)
Smear result of index, n/N (%)				
Positive	178/304 (59)	84/194 (43)	62/76 (82)	32/34 (94)
Negative	126/304 (41)	110/194 (57)	14/76 (18)	2/34 (6)
Drug susceptibility, n/N (%)				
Susceptible	309/335 (92)	207/213 (97)	71/83 (86)	31/39 (80)
Resistant	26/335 (8)	6/213 (3)	12/83 (15)	8/39 (21)
Relationship to index, n/N (%)				
Mother	74/335 (22)	48/213 (23)	16/83 (19)	10/39 (26)
Father	65/335 (19)	23/213 (11)	29/83 (35)	13/39 (33)
Sibling	17/335 (5)	5/213 (2)	8/83 (10)	4/39 (10)
Other family member	113/335 (34)	80/213 (38)	21/83 (25)	12/39 (31)
Non-family member	66/335 (20)	57/213 (27)	9/83 (11)	0/39 (0)
Completion of TPT, n/N (%)				
TPT completed	112/133 (84)	38/45 (84)	70/80 (88)	4/8 (50)
TPT not completed	21/133 (16)	7/45 (16)	10/80 (13)	4/8 (50)

IQR: interquartile range.

Ukrainian refugee children accounted for 8.0% (27/335) of the cohort. Compared with non-Ukrainian participants, they had a significantly increased risk of developing TB (relative risk [RR] 3.4 [95% CI 1.82 to 6.44]), whereas the RR for TBI was not statistically significant.

## Discussion

The proportion of children with TB or TBI in our cohort was higher than in comparable studies,^10–12^ likely due to both enhanced diagnostic strategies and selection bias. Systematic use of chest CT and microbiological testing in high-risk contacts increased case detection from 25 to 39. In addition, nearly one-third of participants were referred to our centre for evaluation of suspected TB, TBI or inconclusive findings.

The age-related increase in TBI risk observed in our cohort is consistent with other studies^12,13^ and may reflect cumulative exposure^14^ and social mobility.^15^ However, in low-incidence countries, a more plausible explanation is the rapid progression of TBI to TB in younger children, supported by the inverse association between age and TB risk.^16,17^

Although we did not demonstrate a protective effect of BCG vaccination in our cohort, several studies have confirmed protection in younger children (up to 5–10 y).^18–20^ Due to the small sample size, we did not stratify participants by age. The development of TB or TBI depends on the balance between exposure intensity, bacterial virulence and the host’s immune status. A substantial proportion of BCG-vaccinated children in our cohort with household exposure developed TB or TBI, indicating that source intensity often outweighs host immunity.

CT was an important diagnostic tool, identifying TB-consistent abnormalities in 29% of children with normal CXR findings, a rate comparable to prior studies.^21,22^ While radiation exposure and the need for anaesthesia in younger children are concerns, CT contributed to a 50% increase in TB detection in our cohort. Microbiological testing also added diagnostic value, confirming TB in cases where imaging was negative or ambiguous and enabling individualized treatment when drug susceptibility patterns differed from presumed index patients.

Treatment adherence in our cohort exceeded European benchmarks for low-incidence settings:^23^ 95.5% of children initiated TPT and 84.2% completed it.

Ukrainian refugee children were at increased risk of TB, largely due to household exposures. Lower social integration and limited participation in contact tracing may partly explain this pattern. To our knowledge, this is the first study to examine the impact of Ukrainian refugee children on TB contact tracing in Europe. Ukrainian refugee children and their caregivers often face considerable cultural and language barriers. Given the higher risk of TB in this population, we consider it essential to provide targeted attention and support. These barriers can be mitigated through the use of professional interpreters, culturally adapted educational materials and the involvement of healthcare professionals with a Ukrainian background, all of which may improve communication, adherence and overall quality of care.

Strengths of the study include its novelty, nationwide recruitment and comprehensive diagnostic approach. Limitations include the modest sample size, loss to follow-up and missing data on ethnicity, socio-economic status and tobacco use—factors that may influence TB risk.

## Conclusions

In this cohort of paediatric TB contacts in the Czech Republic, we observed higher rates of TB and TBI than in comparable studies, likely due to comprehensive evaluation and referral practices. Chest CT and microbiological testing improved diagnostic yield, while younger children showed a greater risk of rapid progression. TPT uptake and completion exceeded international benchmarks, confirming the feasibility of high adherence in low-incidence settings. Ukrainian refugee children emerged as a particularly vulnerable subgroup, underscoring the need for systematic screening, TPT and tailored strategies to strengthen paediatric TB control.

## Supplementary Material

ihaf151_Supplemental_File

## Data Availability

The data underlying this article will be shared upon reasonable request to the corresponding author.
