# Supplementary material for: Determinants of tuberculosis infection and disease in paediatric patients in the Czech Republic after tuberculosis exposure
Source: Int Health. 2025 Dec 22;18(5):767–72. doi: 10.1093/inthealth/ihaf151 (PMC13530277; doi:10.1093/inthealth/ihaf151)
Supplement: Supplementary file 1 [file ihaf151_supplemental_file.docx]

|  |  | **TBI** | | | | | **TB** | | | | | **TBI+TBD** | | | | |
| --- | --- | --- | --- | --- | --- | --- | --- | --- | --- | --- | --- | --- | --- | --- | --- | --- |
|  |  | OR | 95 % CI | B | p-value | OR | | 95 % CI | B | p-value | OR | | 95 % CI | B | p-value |  |
| **Age** |  | **1.121** | **(1.030 - 1.220)** | **0.114** | **0.008** | **0.839** | | **(0.742 - 0.950)** | **-0.175** | **0.005** | 1.037 | | (0.953 - 1.129) | 0.037 | 0.398 |  |
| **Gender** | female | 1.303 | (0.651 - 2.606) | 0.265 | 0.455 | 0.504 | | (0.193 - 1.319) | -0.685 | 0.163 | 0.930 | | (0.464 - 1.863) | -0.073 | 0.837 |  |
| **BCG vaccination status** | yes | 0.852 | (0.362 - 2.005) | -0.160 | 0.714 | 1.258 | | (0.426 - 3.718) | 0.230 | 0.678 | 0.976 | | (0.404 - 2.357) | -0.024 | 0.957 |  |
| **Household exposure** | no | 0.588 | (0.215 - 1.604) | -0.531 | 0.300 | **0.231** | | **(0.054 - 0.994)** | **-1.464** | **0.049** | **0.362** | | **(0.141 - 0.928)** | **-1.017** | **0.034** |  |
| **Smear result of index** | positive | **4.662** | **(1.916 - 11.343)** | **1.539** | **0.001** | **11.351** | | **(2.238 - 57.583)** | **2.429** | **0.003** | **9.487** | | **(3.958 - 22.737)** | **2.250** | **0.000** |  |
| **Drug susceptibility** | resistant | 1.887 | (0.644 - 5.527) | 0.635 | 0.247 | 1.222 | | (0.308 - 4.852) | 0.200 | 0.776 | **4.034** | | **(1.045 - 15.564)** | **1.395** | **0.043** |  |
| **Relationship to index** | mother | **1.000** |  |  | **0.039** | 1.000 | |  |  | 0.353 | **1.000** | |  |  | **0.000** |  |
|  | father | **2.910** | **(1.073 - 7.889)** | **1.068** | **0.036** | 2.592 | | (0.744 - 9.026) | 0.952 | 0.135 | **6.127** | | **(1.970 - 19.058)** | **1.813** | **0.002** |  |
|  | sibling | 3.712 | (0.851 - 16.199) | 1.312 | 0.081 | 5.285 | | (0.762 - 36.638) | 1.665 | 0.092 | **12.367** | | **(1.862 - 82.142)** | **2.515** | **0.009** |  |
|  | other family member | 1.568 | (0.488 - 5.030) | 0.449 | 0.450 | 0.969 | | (0.205 - 4.570) | -0.032 | 0.968 | 1.307 | | (0.439 - 3.892) | 0.268 | 0.631 |  |
|  | non-family member | 0.523 | (0.114 - 2.406) | -0.648 | 0.405 | 0.000 | |  | -18.406 | 0.997 | 0.410 | | (0.102 - 1.648) | -0.891 | 0.209 |  |
| Constant |  | **0.031** |  | **-3.485** | **0.000** | **0.111** | |  | **-2.196** | **0.039** | **0.106** | |  | **-2.244** | **0.002** |  |

**Supplements: Results of binary logistic regression for the groups tuberculosis infection, tuberculosis, and tuberculosis infection plus tuberculosis. Statistically significant results are highlighted in bold.**

BCG - bacille Calmette-Guérin, OR – odds ratio, TBI - tuberculousis infection, TB - tuberculousis disease, CI – confidence interval
